# Silodosin as a Novel Inhibitor of Acetylcholinesterase, Butyrylcholinesterase, and BETA-Secretase 1: In Vitro and In Silico Studies

**DOI:** 10.1021/acsomega.5c07084

**Published:** 2025-10-09

**Authors:** Deyse B. Barbosa, Lucas Matheus G. de Oliveira, Géssica O. Mendes, David B. Costa Junior, Tiago A. de Oliveira, Michel P. da Silva, Laila Cristina M. Damázio, Eduardo H. B. Maia, Daniel Luciano Falkoski, Isabela F. de S. Marra, Marcelo S. Valle, Alisson M. da Silva, Alex G. Taranto, Victor D. A. da Silva, Paulo B. de Carvalho, Franco Henrique A. Leite

**Affiliations:** † Postgraduate Program in Biotechnology, State University of Feira de Santana, Transnordestina Avenue, Novo Horizonte, Feira de Santana, Bahia 44036-900, Brazil; ‡ Laboratory of Chemoinformatics and Biological Evaluation, Health Department, State University of Feira de Santana, Transnordestina Avenue, Novo Horizonte, Feira de Santana, Bahia 44036-900, Brazil; § Laboratory of Neurochemistry and Cell Biology, Department of Biochemistry and Biophysics, 28111 Federal University of Bahia - Institute of Health Sciences, Reitor Miguel Calmon Avenue, Salvador, Bahia 40110-902, Brazil; ∥ Laboratory of Bioinformatics and Drug Design, Federal University of São João del-Rei, 74 Dom Helvécio Square, Dom Bosco, São João del-Rei, Minas Gerais 36301-160, Brazil; ⊥ Computer Science Department, Federal Center of Technological Education of Minas Gerais, 400 Álvares de Azevedo Street, Bela Vista, Divinópolis, Minas Gerais 34403-822, Brazil; # Feik School of Pharmacy, 130378 University of the Incarnate Word, 703 E Hildebrand Avenue, San Antonio, Texas 78212, United States; ¶ Laboratory of Rehabilitation, Department of Medicine, Federal University of São João del-Rei, 80 Padre João Pimentel Street, São João del-Rei, Minas Gerais 36301-158, Brazil; ∇ Laboratory of Organic Synthesis, Department of Natural Sciences, Federal University of São João del-Rei, 80 Padre João Pimentel Street, São João del-Rei, Minas Gerais 36301-158, Brazil

## Abstract

Alzheimer’s Disease (AD) is a progressive neurodegenerative disorder and the leading cause of cognitive decline in older adults. Several biomarkers of AD have been identified, but its pathogenesis has not yet been completely elucidated. One of the most relevant hypotheses proposed to explain the cognitive impairment caused by this disease is the cholinergic hypothesis, which postulates that loss of cholinergic neurons is one of its causes and that the subsequent reduction of acetylcholine levels in the synaptic cleft can be compensated through the inhibition of acetylcholinesterase (AChE) and butyrylcholinesterase (BuChE). Another well-known hypothesis is the amyloid-beta hypothesis, which explains the disease as being caused by the formation and accumulation of amyloid plaques in a cascade of enzymatic events starting with the cleavage of an amyloid precursor protein (APP) by beta-secretase 1 (BACE-1). Previous studies have shown that silodosin has the structural requirements for the inhibition of those three enzymes (AChE, BuChE, and BACE-1), which suggests that it can be useful as a multitarget candidate to treat Alzheimer patients. This study aims to assess the effect of silodosin on cellular viability, measure the inhibitory activity against AChE, BuChE, and BACE-1, and evaluate the molecular behavior of all three inhibitor–enzyme systems by molecular dynamics (MD) simulations. Cell viability assays through the MTT method showed that silodosin concentrations of less than 10 μM are safe to be used. Enzymatic assays revealed AChE inhibitory activity at high micromolar levels (IC50 >500.0 μM) but inhibited BuChE at low micromolar levels (IC50 = 3.02 ± 0.05 μM). BACE-1 inhibition assays have shown significant reduction at three micromolar. MD simulations demonstrated that silodosin promotes late stabilization of the AChE complex, but the simulations involving BuChE and BACE-1 revealed that the compound promotes system stabilization at early stages and has the structural requirements to inhibition.

## Graphical abstract

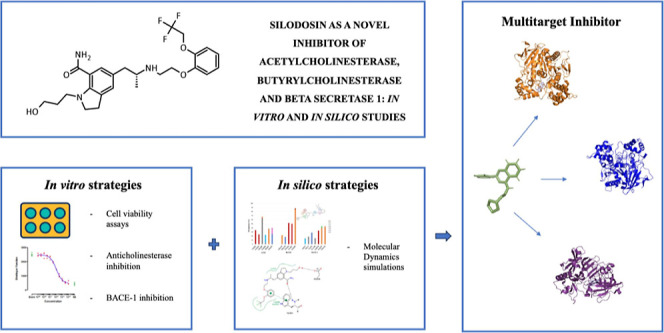

## 1 Introduction

Alzheimer’s disease (AD) is a chronic neurodegenerative pathology, characterized by cognitive decline and behavioral changes.^1^ It is the most common form of dementia worldwide and is the main reason responsible for cognitive impairment in the elderly.^2^

The initial symptoms for AD are usually short-term memory loss, apathy, and depression; as the condition evolves, communication problems, confusion, behavioral changes, gait disturbances, and difficulty in swallowing are observed.^3^ However, the characteristic symptoms of the disease are only manifested years after the appearance of the pathophysiological changes at the neuronal level, which are related to brain tissue atrophy, accumulation of amyloid protein, and cell death,^3–6^ aspects that are considered AD markers.

Despite the identification of several biomarkers for the characterization of AD, its pathogenesis has not yet been fully elucidated.^7^ More than 95% of AD cases are not determined by genetic conditions but are related to a series of etiopathogenic mechanisms which characterize AD as a multifactorial disease.^8,9^ Several hypotheses have been proposed to explain the origins and symptoms of this disease, of which the cholinergic hypothesis and the amyloid hypothesis are considered the most important.^10^

The cholinergic hypothesis is the oldest hypothesis, which explains the symptoms and progression of the disease as being caused by the reduction of acetylcholine levels in the brain due to the degeneration of cholinergic neurons. Cholinesterases—acetylcholinesterase (AChE, E.C. 3.1.1.7) and butyrylcholinesterase (BuChE, E.C. 3.1.1.8)—are enzymes responsible for the hydrolysis of acetylcholine present in the synaptic cleft.^11^ The inhibition of cholinesterases has shown positive results in the treatment of individuals with AD.

The amyloid hypothesis, in turn, relates AD to the formation of extracellular deposits of the β-amyloid peptide (βA), produced by the action of proteolytic enzymes (alpha, beta, and gamma secretase) from the amyloid precursor protein (APP), with beta-secretase 1 (BACE-1, E.C. 3.4.23.46) being the most involved in the production of those deposits.^12,13^ BACE-1 is responsible for hydrolyzing APP into peptide fragments, which undergo further action by gamma secretase and then form the βA peptide. The aggregation of these peptides promotes the formation of structures that are deposited in the extracellular environment of the neuronal tissue and collaborates to the installation of a progressive synaptic dysfunction, neurodegeneration, and neuronal death.

Despite the impact of AD in public health, the resources available for treating AD patients are limited and solely based on palliative control, presenting low efficacy and serious adverse side effects.^3,14^ With the discovery of the role of BACE-1 in the progression of AD, the pharmaceutical industry has dedicated efforts to identifying BACE-1 inhibitors but without success as they, for the most part, failed clinical trials and presented no significant decrease of cognitive impairment.^15,16^ In addition to the search for micromolecules, a widely used approach consists of the development of monoclonal antibodies targeting different stages of an amyloid cascade (since monomers to installed plaques),^17^ due to benefits associated with selectivity and higher half-life.

Recently, regulatory agencies have approved biopharmaceutical products targeting AD pathophysiology; however, one of them was removed from market due to concerns about its safety and the validation of the effectiveness of the ones that are still being used is not yet available.^18–21^ Studies are trying to understand the causes of those failures, but they seem to stem from the “one target approach”, considering only one of the pathological ways and neglecting the multifactorial aspect of AD,^8,18^ which reinforces the need for treatments that consider the complexity of AD and are capable of modulating more than one target simultaneously. The concept of a disease involving multiple biological pathways has spurred the pharmaceutical industry on studying the development of multitarget drugs^22,23^ with positive results.

Despite their potential benefits, the development of multitarget drugs is a challenging task.^24^ Besides all the hurdles, a new drug must clear before it reaches the pharmaceutical market, a process that can take over ten years, and multitarget compounds also need to achieve a balance between their activity against the targets of interest, their selectivity and an appropriate pharmacokinetic profile.^25^ To expedite results, the pharmaceutical industry has been taking advantage of drug repositioning as an alternative way to make the process easier, where drugs already approved by regulatory agencies for known diseases are repurposed for new indications.^26^

Thereby, advances in technology, artificial intelligence algorithms, and the development of bioinformatics and in silico tools have made it possible to obtain more accurate insights into potential interactions between drugs and protein targets.^27,28^ The main in silico strategies for drug repositioning are structure-based approaches, either through ligand-based virtual screening, such as pharmacophore modeling, or through target-based virtual screening, such as molecular docking.

Based on those facts, a previous study has used in silico strategies for drug repositioning and has shown that ZINC3806063, also known as silodosin—an FDA-approved drug, orally administered to treat benign prostatic hyperplasia—has structural requirements suggesting it can be useful as a multitarget inhibitor to treat AD.^29^ This compound consists of a substituted indoline group and an *O*-alkylated catechol linked by a flexible six-atom chain. This study aims to identify the activity of this potential inhibitor against cholinesterases and BACE-1 and evaluate the molecular behavior of those inhibitor–enzyme systems by molecular dynamics simulations, as described in Scheme 1 and detailed in the following sections.

**Scheme 1:**
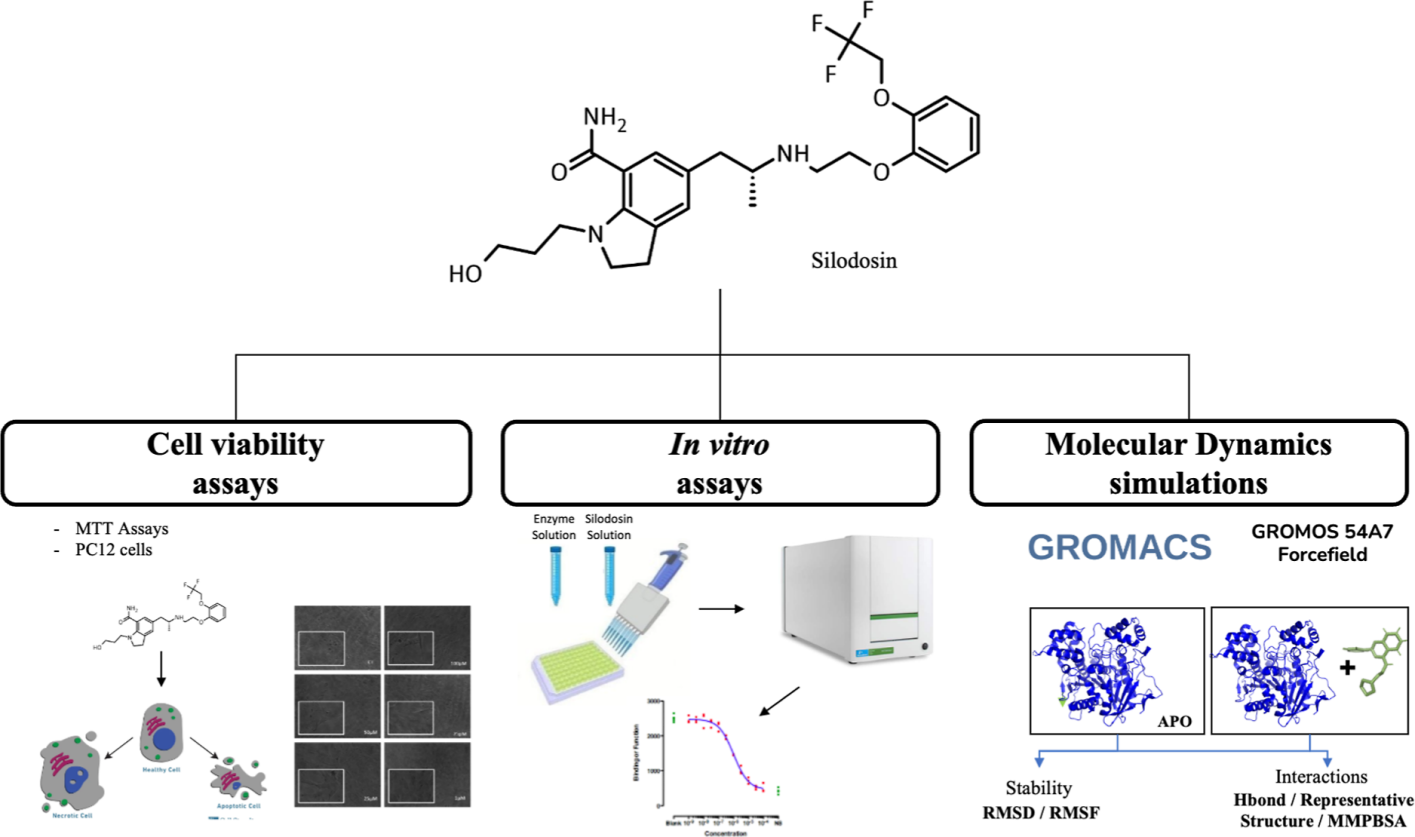
Methodology Steps to Silodosin Evaluation and Molecular Dynamics Simulation

## 2 Materials and Methods

### 2.1 Determination of Cell Viability

PC12 cells were cultured in DMEM high glucose (Gibco) supplemented with 3.7 g/L sodium bicarbonate (Sigma), 1% antibiotic streptomycin (100 mg/mL) and penicillin (100 U/mL), and 1% FBS and maintained in an incubator with a humidified atmosphere of 5% CO_2_ at 37 °C until 80% confluence. Cells were then detached using a solution of trypsin 0.25% with EDTA 0.2% and plated in a 96-well plate at a density of 7 × 10^4^ cells per cm^2^. After 24 h incubation, PC12 cells were incubated with silodosin at different concentrations (1, 10, 25, 50, 75, or 100 μM) for 24 h without FBS at 37 °C in an incubator with a humidified atmosphere of 5% CO_2_. On the next day, the medium was removed and replaced by a medium containing 3-(4,5-dimethylthiazolyl-2)-2,5-diphenyltetrazolium bromide (MTT) in a concentration of 0.5 mg/mL and incubated for 2 h at 37 °C. After that time, DMSO (200 μL) was added to the well and homogenized for 15 min in the dark, and the Optical Density (OD) was read at 590 nm wavelength (Varioskan LUX multimode microplate reader). The cell viability rate was calculated as follows: Cell viability rate (%) = (OD_test group_ – OD_blank of test group_)/(OD_control group_ – OD_blank of control group_) × 100%.^30,31^ Data were analyzed using GraphPad Prism software, using the nonparametric Kruskal–Wallis test followed by Dunnet’s multiple comparison post-test. Data are expressed as the mean ± SEM from three independent experiments (*N* = 3).

### 2.2 Cellular Assays to Determine In Vitro Beta-Secretase Inhibition

The evaluation of BACE-1 inhibitory activity was performed using the beta-secretase activity fluorometric assay kit (MAK237) obtained from Sigma-Aldrich according to the instructions from the manufacturer. Cells were placed in a 6-well plate in a confluence of 5.2 × 10^5^ cells per cm^2^ for 24 h in an incubator with a humidified atmosphere of 5% CO_2._ and then treated with DMSO (control) or 3 μM of Silodosin for another 24 h. On the other day, cells were detached from the 6-well plate using the above-mentioned solution with trypsin and EDTA and centrifuged for 5 min at 700*g*. The supernatant was discarded, and 0.1 mL of ice-cold extraction buffer was added to the cell pool. The cell lysate was incubated on ice for 10 min and centrifugated at 10000*g* for 5 min at 4 °C, and the supernatant was transferred to a new tube and kept on ice. To each well in a 96-well plate was added 50 μL of cell lysate. Cell lysate from the well treated with DMSO was treated as a negative and positive control (with the addition of 2 μL of the BACE-1 standard inhibitor from the kit). 50 μL of the 2× Reaction Buffer was added and then the samples were gently mixed and incubated for 20 min at 37 °C. After the incubation time, 2 μL of the substrate was added and the plate was incubated for 10 min at 37 °C. The plate was covered and gently mixed and incubated in the dark at 37 °C for 1 h and then the samples were read with a fluorescence plate reader (ƛ_ex_ = 355 nm and ƛ_em_ = 510 nm). Total protein concentration was assessed using the Lowry method.^32^ BACE-1 activity was measured by fluorescence units per μg of protein in the sample, normalizing the results to protein content to account for variations in the sample concentration. Statistical analyses were realized following the same steps described in section 2.1.

### 2.3 Enzymatic Assays to Determine In Vitro Anticholinesterase Activity

The evaluation of anticholinesterase activity was performed as previously described by Ellman and co-workers,^33^ adapted to 96-well microplates, using kinetic parameters previously determined by our group.^34^

The enzymes utilized were the acetylcholinesterase from Electrophorus electricus type IV and butyrylcholinesterase obtained from equine serum; Ellman’s reagent (5.5′dithiobis-2-nitrobenzoic acid—DTNB), albumin from bovine serum (BSA), acetylthiocholine (ACTI), and silodosin were also used. All of those materials were obtained from Sigma-Aldrich. A Multiskan FC microplate reader was used for spectrophotometric evaluation and was obtained from Thermo Scientific. For the AChE assays, in each well were added 140 μL of 0.1 M phosphate buffer at 7.4 pH containing 0.1% BSA, 20 μL of the sample diluted in ethanol, 20 μL of enzyme solution at 0.15 U/mL, 10 μL of 0.01 M DTNB, and 10 μL of 0.125 mM ACTI, similar to what was described by de Almeida and co-workers.^34^ For BuChE assays, in turn, 20 μL of the enzyme solution at 0.10 U/mL and 10 μL of 0.744 mM ACTI were added to the other reactional compounds.

Silodosin, as well as other reagents, was obtained from Sigma-Aldrich and was tested at concentrations between 0.25 and 500 μM to calculate the IC50 value. Ethanol was used as a diluent to the sample and also as a negative control. Eserine (10 μM) was used as the standard inhibitor in both assays. The tests were realized in triplicate; the absorbance was measured at 405 nm in cycles during 10 min for AChE and 20 min for BuChE.

### 2.4 Molecular Dynamics

APO forms and complexes with silodosin were analyzed by Molecular Dynamics (MD) simulations. The silodosin 3D coordinates were submitted to the ATB 3.0 server^35^ for its topology generation. The parameters of atomic charge, bond length, torsional angles, and dihedrals were obtained using the GROMOS96 54A7 force field.^36^ The MD simulations were performed in the GROMACS 5.1.2 package^37^ in which we adopted the GROMOS96 54A7 force field parameters, with 25 °C as temperature and pressure of 1 atm.

The 3D structures of AChE, BuChE, and BACE-1 were obtained from the PDB (ID AChE: 4M0E; ID BuChE: 4BDS; ID BACE-1:6UWP), from which the crystallographic ligand, water molecules, and artifacts were removed. Nonmodeled regions were built through the SWISS-MODEL server.^38^

The protonation state was adjusted in the pdb 2gmx module implemented in GROMACS 5.1.2 according to pH 7.4 for cholinesterases^39^ and pH 4.5 for BACE-1.^40^ The residue p*K*_a_ values were evaluated on the H++ server (http://biophysics.cs.vt.edu/index.php). A dodecahedral box with water model SPC-E^41^ was used to solvate the systems with a minimum distance of 1.4 nm from the box edges. For neutralization, in systems involving AChE (APO and complex), 7 Na^2+^ ions were added, while in systems with BuChE and BACE-1, 4 Cl^–^ ions were added.

APO and complex systems were minimized in two steps: initially by the Steepest Descent (SD) algorithm with 10,000 cycles and, later, by the Conjugated Gradient (GC) algorithm with 1000 cycles. After the minimization steps, the equilibration step was performed (*t* = 1 ns) and, finally, the production dynamics was performed under 300 K and 1 atm. The time of production dynamics varied considering aspects of each system: AChE_APO_ = 100 ns; AChE_complex_ = 200 ns; BuChE_APO_ = 150 ns; BuChE_complex_ = 100 ns; BACE-1_APO_, and complex = 100 ns. GROMACS modules (rms, rmsf, and hbond functions) were utilized to analyze the stability and behavior of each system. Binding free energy of complexes was calculated by the molecular mechanics Poisson–Boltzmann Surface Area method (MM/PBSA) (g_mmpbsa tool).

## 3 Results and Discussion

Drug repositioning has emerged as a prominent strategy in pharmaceutical innovation and research, as it enables the identification of new therapeutic uses for existing drugs beyond their original indication.^42^ Through this approach, a previous study^43^ has used in silico strategies to prioritize novel multitarget drug candidates to DA treatment and identify silodosin—an FDA-approved drug that is used to treat patients with lower urinary tract symptoms and benign prostate hyperplasia^44^—as a potential triple inhibitor for AChE, BuChE, and BACE-1. In this paper, Barbosa and co-workers (2023) built and validated a pharmacophore model with structural requirements to triple inhibition, which suggest that multitarget ligands must have hydrophobic centers, H-bond acceptors, and a positively charged center. This pharmacophore model was used for virtual screening (QFIT_silodosin_ = 31.67), followed by molecular docking screening (Score_AChE_ = −8.0 kcal/mol; Score_BuChE_ = −8.1; Score_BACE‑1_ = 43.55), together with physical–chemical parameter prediction. In silico evaluation of silodosin demonstrated that this compound has structural requirements described as triple inhibition. However, in silico data are not enough to prove biological activity and, for this reason, in vitro tests were realized to confirm biological properties.

### 3.1 Determination of Cell Viability

The characterization of potential development of neurotoxic risk consists of an important challenge in drug development.^45^ Although in vivo studies can provide useful data, they require extensive resources such as animals, researchers, time, and money. On the other hand, in vitro tests are relatively fast and a less demanding way to test chemicals for their neurotoxic properties.^46^ For neurodegeneration studies, the effects of chemical compounds can be evaluated using cell lines derived from rodents or humans. Among these, rat pheochromocytoma PC12 cells are widely utilized due to their ease of culture, high proliferation capacity, and ability to differentiate into neuron-like cells in response to the nerve growth factor.^47,48^ These characteristics make PC12 cells a valuable model for studying AD.^49,50^

For the in vitro test, the first step was to determine silodosin toxicity in the cell system. For this, we performed an MTT viability assay in PC12 submitted to silodosin in different concentrations. Silodosin has a dose-dependent response in PC12 cell lines. 7.5 μM silodosin was not able to significantly reduce cell viability (Figure 1), but higher concentrations showed a toxic or cytostatic effect.

**Figure 1:**
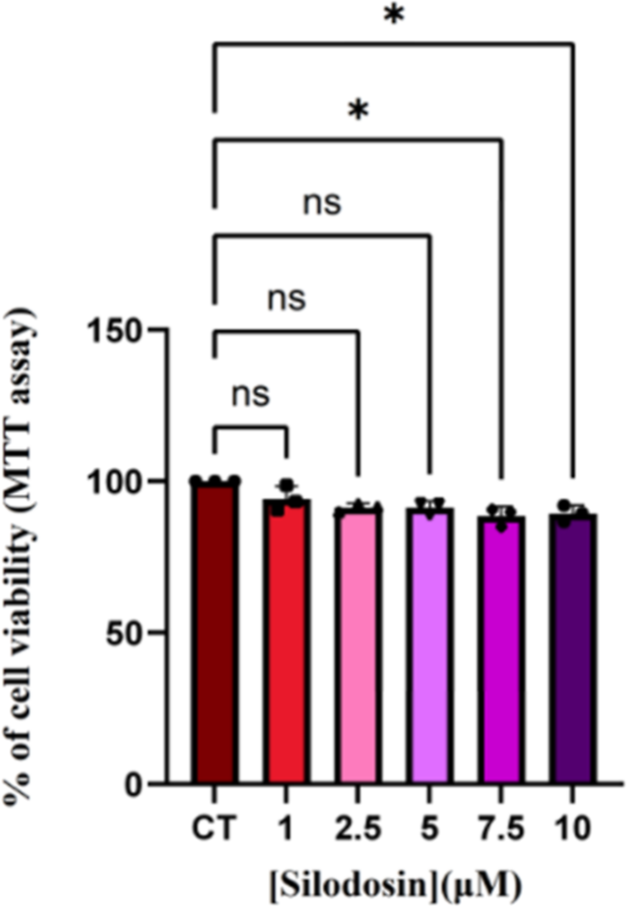
Silodosin effect at PC12 cells determined with MTT. Calculated percentage of cell viability in PC12 cells at control conditions (CT) and treated with silodosin at different concentrations. Data are expressed as mean ± SEM *N* = 3. ns = not statistically significant; *p <0.05.

### 3.2 Cellular Assays to Determine In Vitro Beta-Secretase Inhibition

PC12 cell lines were used to test the effect of Silodosin on BACE-1 activity. Those cells can express BACE-1 that are responsible for the secretion of neurotransmitters and regulation of exocytosis.^51,52^ The assay kit that was used to evaluate BACE-1 activity provided a fluorescence method for detecting BACE-1 activity in samples of the total protein extract from PC12 treated with silodosin or DMSO as a negative control. This kit is validated for qualitative purposes, with relative inhibition in comparison to the negative control. The kit includes a proprietary reference inhibitor, which is used as a positive control. The assay measures the level of secretase enzymatic activity proportionally to the level of fluorescence activity.

In this study, it was possible to observe that silodosin at 3 μM reduced BACE-1 relative activity when compared to the negative control with values almost equal to the sample treated with a standard inhibitor (Figure 2). This result highlights that silodosin has anti-BACE-1 activity at a concentration considered safe according to cell viability tests and, to evaluate its multitarget potential, in addition to the cellular assays to evaluate anti-BACE-1 activity, silodosin was evaluated against cholinesterases.

**Figure 2:**
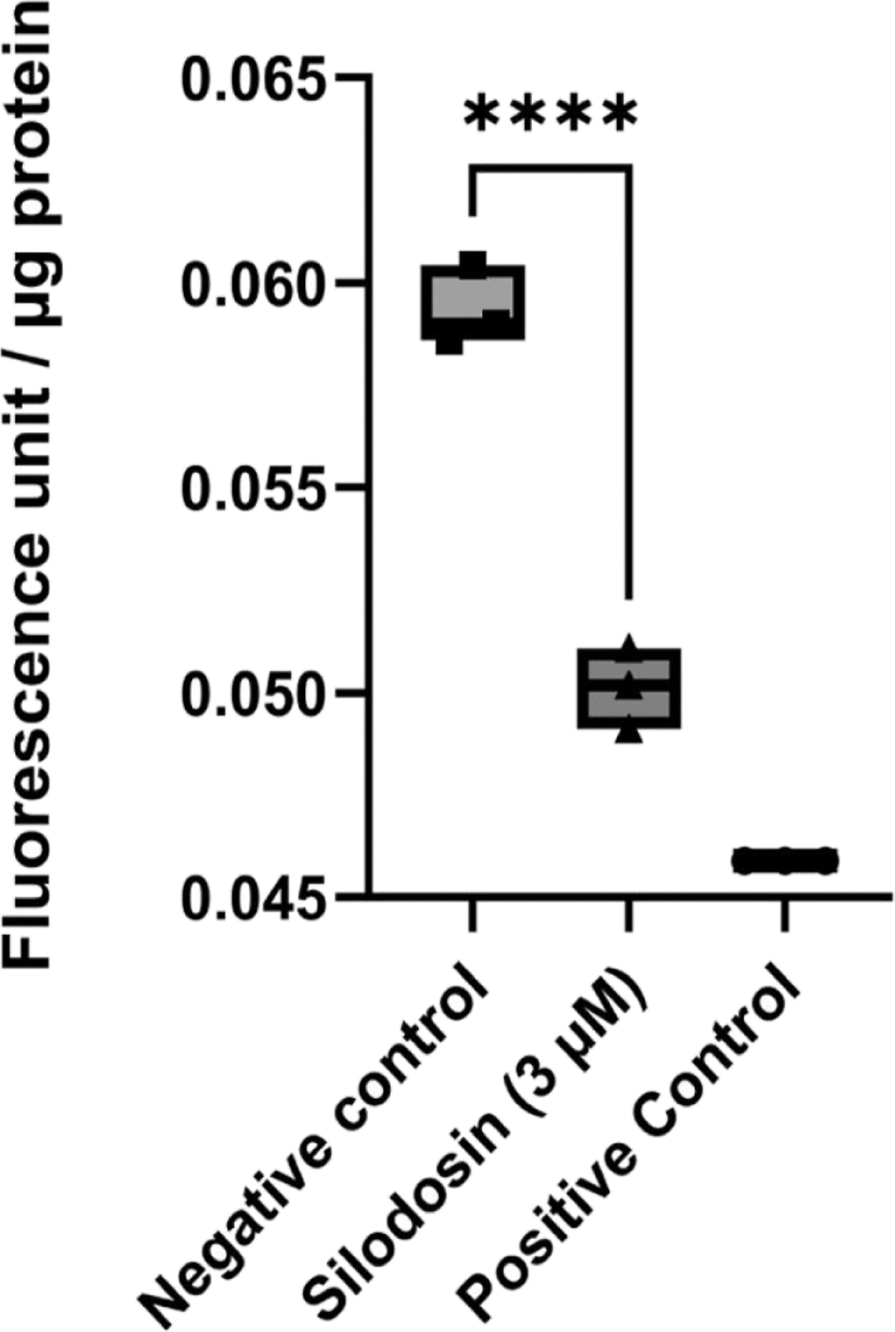
Silodosin effect (3 μM) at BACE-1 enzymatic activity in PC12 cells determined by the cellular assay (KIT MAK 237). Negative control: protein extract from PC12 cells treated with 0.03% DMSO for 24 h + substrate. Silodosin group: protein extract from PC12 cells treated with 3 μM silodosin for 24 h + substrate. Positive control: protein extract from PC12 cells treated with 0.03% DMSO for 24 h + BACE1 inhibitor + substrate. Data are expressed as mean ± SEM (*n* = 3).

### 3.3 Enzymatic Assays to Determine In Vitro Anticholinesterase Activity

The enzymatic assays revealed that silodosin has a low ability to inhibit AChE activity (IC50 >500.0 μM, data not shown) while showing a satisfactory effect on BuChE inhibition (IC50 = 3.02 ± 0.05 μM, Figure 3).

**Figure 3:**
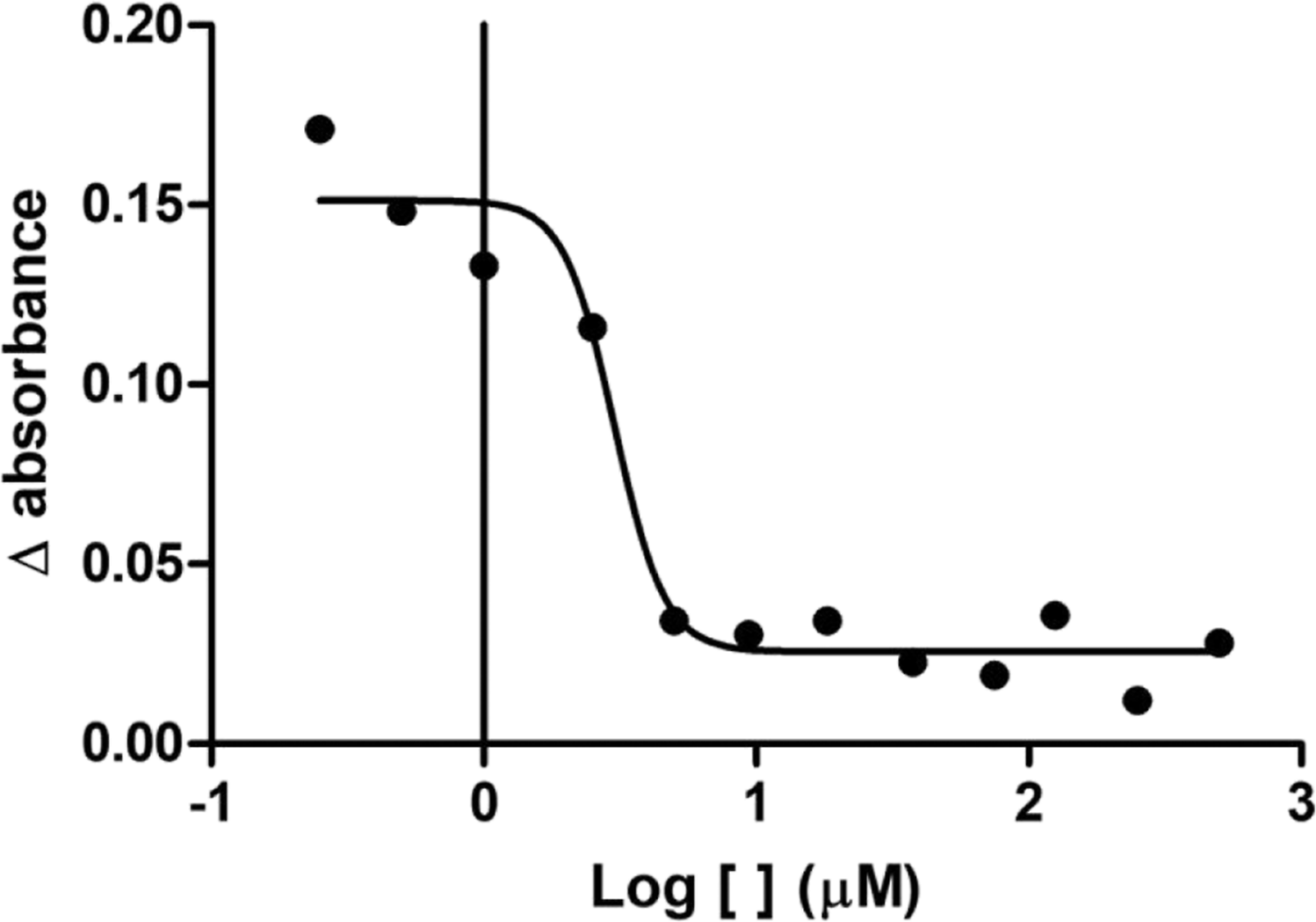
Concentration–response curve of the in vitro antibutyrylcholinesterasic effect for silodosin (*n* = 3).

IC50 calculation suggests that reduced concentrations of silodosin can inhibit the catalytic activity of BuChE, despite its low affinity for AChE. This result is similar to what was observed in previous dual inhibition studies, where tested molecules have a better inhibition profile against BuChE.^53–57^ This fact can be explained by the volume of the active site, which in AChE is relatively small (302 Å^3^) compared to the BuChE site (502 Å^3^).^58^ Additionally, the AChE site is lined with aromatic residues, which reduces the available space for inhibitors to accommodate, when compared to BuChE (Figure 4). These characteristics may confer silodosin selectivity for BuChE over AChE.

**Figure 4:**
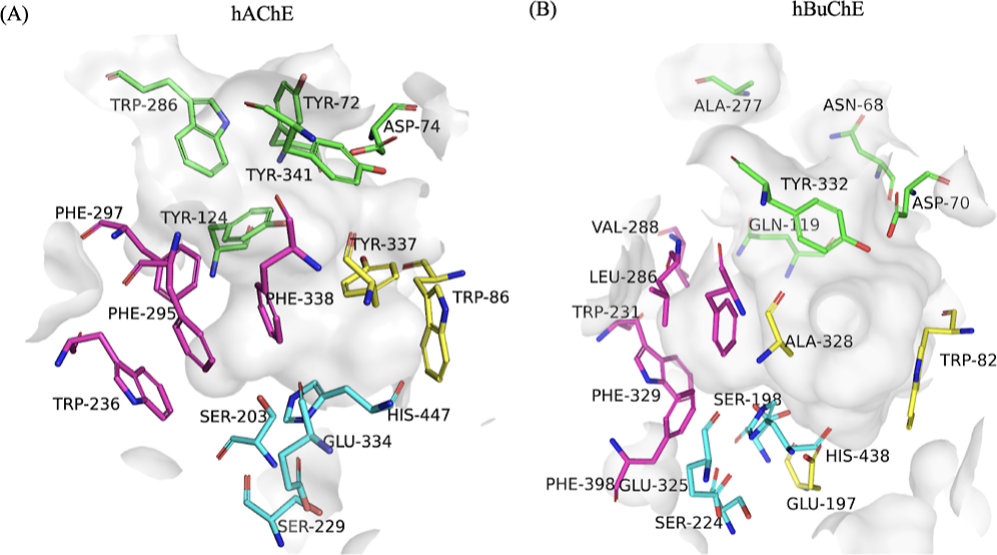
Active site of (A) AChE (PDB ID: 4M0E) and (B) BuChE (PDB ID: 4BDS). The gorge surface is represented in semitransparent gray. The key residues are shown as sticks. The catalytic residues are in cyan; the anionic site residues are in magenta; the choline-binding site residues are highlighted in yellow; and the peripheral site residues are in green.

### 3.4 Molecular Dynamics

Molecular dynamics simulations allow the evaluation of the molecular behavior as a function of time from an atomistic point of view, considering secondary structure and side-chain orientation from both ligand and macromolecule.^59,60^ Those simulations consider not only the ligand as flexible but also the protein, and this approach can play an effective role in identifying false positives obtained by molecular docking.^61^ In this study, those simulations evaluated the stability of silodosin in the active site of the three enzymes (AChE, BuChE, and BACE-1), the intermolecular interactions that are responsible for it, and thermodynamic parameters involving complexes.

In order to evaluate systems stability during MD simulations, the Root Mean Square Deviation (RMSD) was initially analyzed, a metric that evaluates the structural stability of the system through the analysis of conformational changes in the protein structure in comparison to the starting structure.^62^ MD simulations with the complexes (Silodosin bound to AChE, BuChE, and BACE-1) and the APO forms of the proteins had their RMSD for the main chain (backbone) along the trajectory (Figure 5).

**Figure 5:**
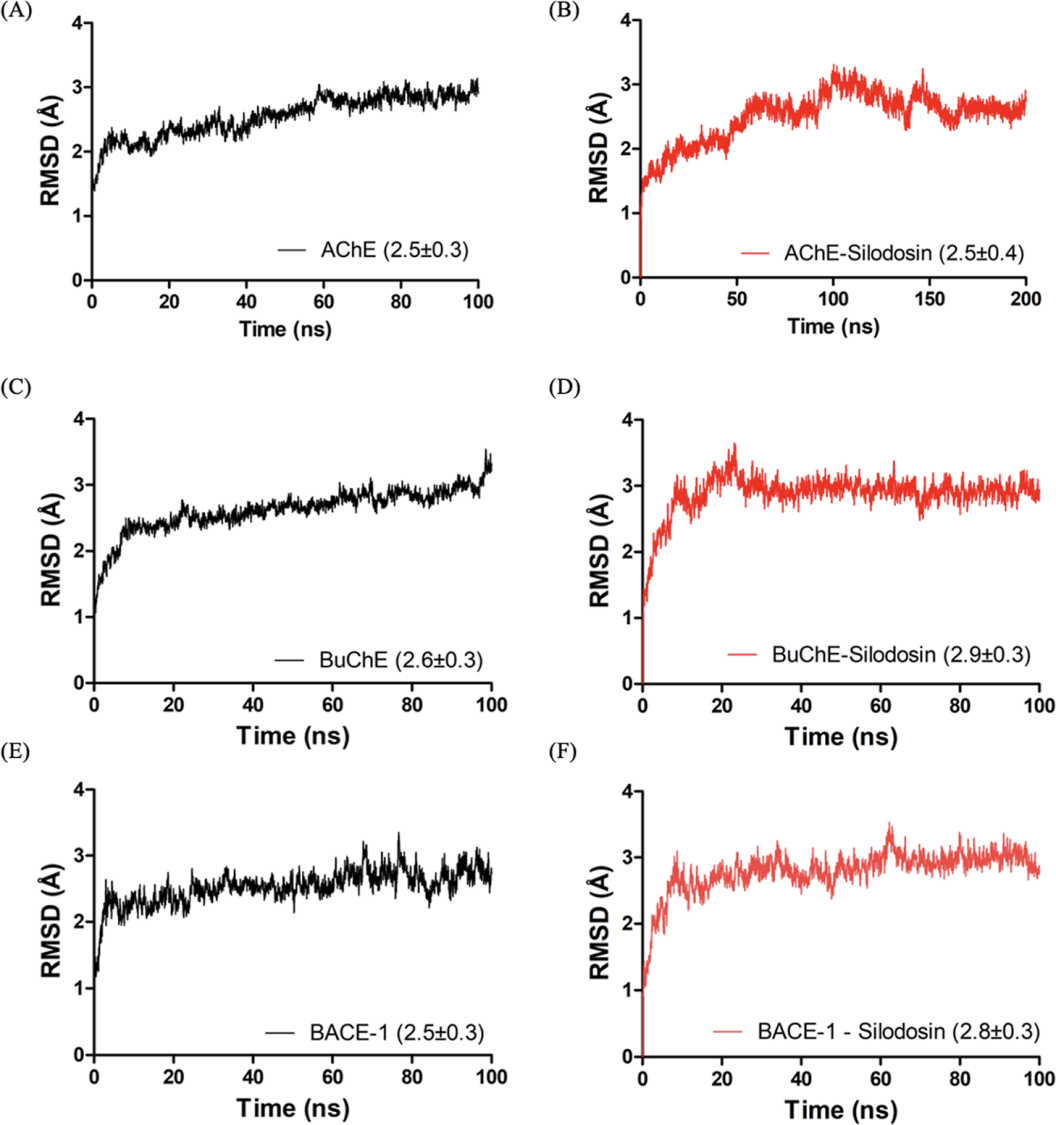
RMSD (backbone) of structures of AChE (A—APO form; B—complex), BuChE (C—APO form; D—complex), and BACE-1 (E—APO form; F—complex) during MD simulations.

The evaluation of RMSD also enables the identification of specific time steps in the trajectory at which the system reaches equilibrium during conformational changes.^62^ In most cases, it is possible to observe the occurrence of a progressive increase in the RMSD value in the initial stages until the system stabilizes, with variations of RMSD below 3.0 Å being considered adequate.^59,63^ It was possible to observe in these simulations the occurrence of an equilibrium before achieving 100 ns of simulation in the systems containing the proteins in their APO form for AChE (Δ*t* = 40 ns) and for BACE (Δ*t* = 60 ns) but not for BuChE, for which was necessary to extend until 150 ns (Δ*t* = 40 ns). In the systems containing the ligand, in turn, it reached early equilibrium for BuChE (Δ*t* = 75 ns) and for BACE-1 (Δ*t* = 35 ns). For the system containing AChE complexed to silodosin, 100 ns was not enough time to observe the stabilization period. For this reason, simulation time was extended to 200 ns, and it showed that the system reaches equilibrium at the simulation time of 165 ns (Δ*t* = 35 ns). This result can probably explain what was observed in the inhibition test, in which the assayed molecule was not able to properly inhibit the enzymatic activity, which suggests that it was possibly due to its inability to establish interactions strong enough to stabilize the system in the early stages.

RMSD considers the entire structure of the protein, which represents important information. However, this information does not guarantee stability since it does not consider the fluctuation of specific regions of the protein as the binding site. In addition to the RMSD, atomic fluctuations can be evaluated individually by calculating the Root Mean Square Fluctuation (RMSF). For this reason, fluctuation plots of the residues for the APO form were generated and compared with those of the respective complexes with silodosin, during the productive phase (Figures 6, 7 and 8).

**Figure 6:**
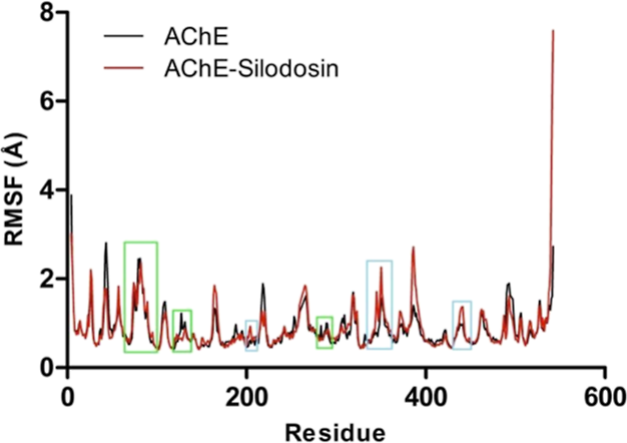
RMSF (Å) (backbone) from the APO and complex of AChE with silodosin during the productive phase (Δ*t* = 35 ns). Green highlights correspond to regions that comprehend peripheral site residues. Cyan highlights correspond to regions that comprehend catalytic residues.

**Figure 7:**
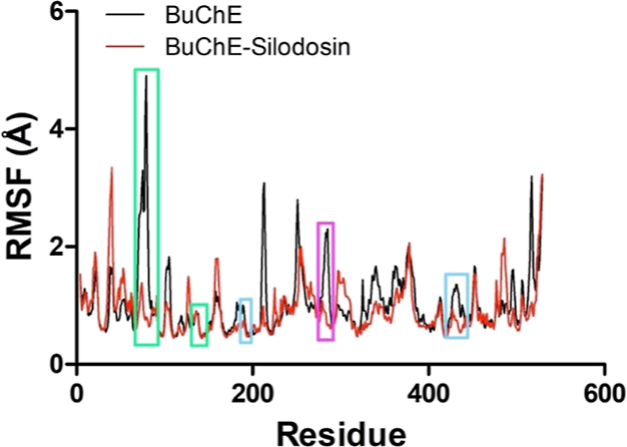
RMSF (Å) (backbone) from the APO and complex of BuChE with silodosin during the productive phase (Δ*t* = 75 ns). Green highlights correspond to regions that comprehend peripheral site residues. Magenta highlights correspond to regions that comprehend anionic site residues. Cyan highlights correspond to regions that comprehend catalytic residues.

**Figure 8:**
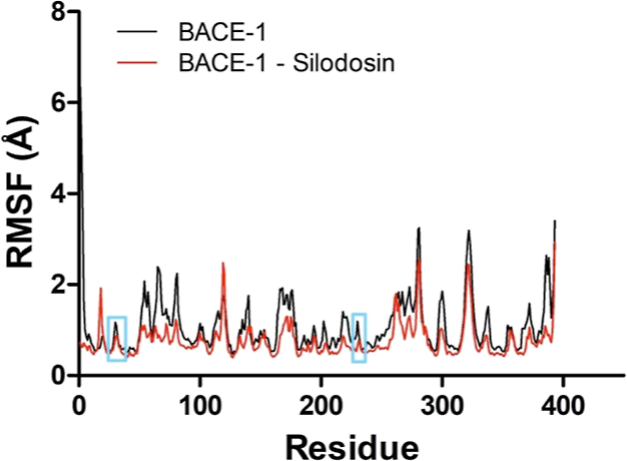
RMSF (Å) (backbone) from the APO and complex of BACE-1 with silodosin during the productive phase (Δ*t* = 35 ns). Cyan highlights correspond to regions that comprehend catalytic residues.

Data reveal that the atomic fluctuation of the complex with AChE (RMSF = 0.9 ± 0.5 Å) is statistically similar to APO form fluctuations (RMSF = 0.8 ± 0.4 Å; Figure 6). The active site regions followed a similar fluctuation profile in both the complexed and the APO forms, which suggests that ligand presence does not promote significant conformational changes of the protein structure.

On the other hand, atomic fluctuations of the BuChE APO form (RMSF = 1.0 ± 0.5 Å) and complex (RMSF = 0.9 ± 0.4 Å) suggest that the second system is more stable (Figure 7). Such consideration is evidenced when observing the regions of the active site highlighted in the graph in which the system without ligand presents more evident fluctuations. This data seems to be aligned with the in vitro test results, since conformational changes of the active site are primordial to catalysis.^64^

In the BACE-1 APO form (RMSF = 1.1 ± 0.4 Å) and complex (RMSF = 0.7 ± 0.4 Å), the effects of the ligand on atomic fluctuation are even more pronounced, suggesting greater stability for the complexed form (Figure 8). The region of catalytic residues presents low fluctuation in both systems, which demonstrates that there were no significant changes in the conformation of the active site in the simulations. However, it is possible to observe a greater fluctuation in the region that comprehends residues Val67 to Glu77 in the APO system compared to the complexed system. This behavior suggests that silodosin performs interactions that possibly prevent the opening of the flap region and inhibit the catalytic activity of BACE-1, similar to what was observed in previous studies.^65^

RMSD and RMSF data demonstrate that silodosin has molecular characteristics that allow stabilization of the structures of AChE, BuChE, and BACE-1, despite the late stabilization of AChE systems. The stabilization arises from intermolecular interactions that are established between ligand and protein residues, and those interactions yield to a biological response. In this context, the hydrogen bonds are considered important in biology because of its strength that is enough to create a state of complex between two molecules.^66^ Since those interactions are transitory, they are analyzed considering their permanence during simulation time and interactions with permanence longer than 10% of simulation (Figure 9), and in this case, we considered the productive phase the last 35 ns from each simulation.

**Figure 9:**
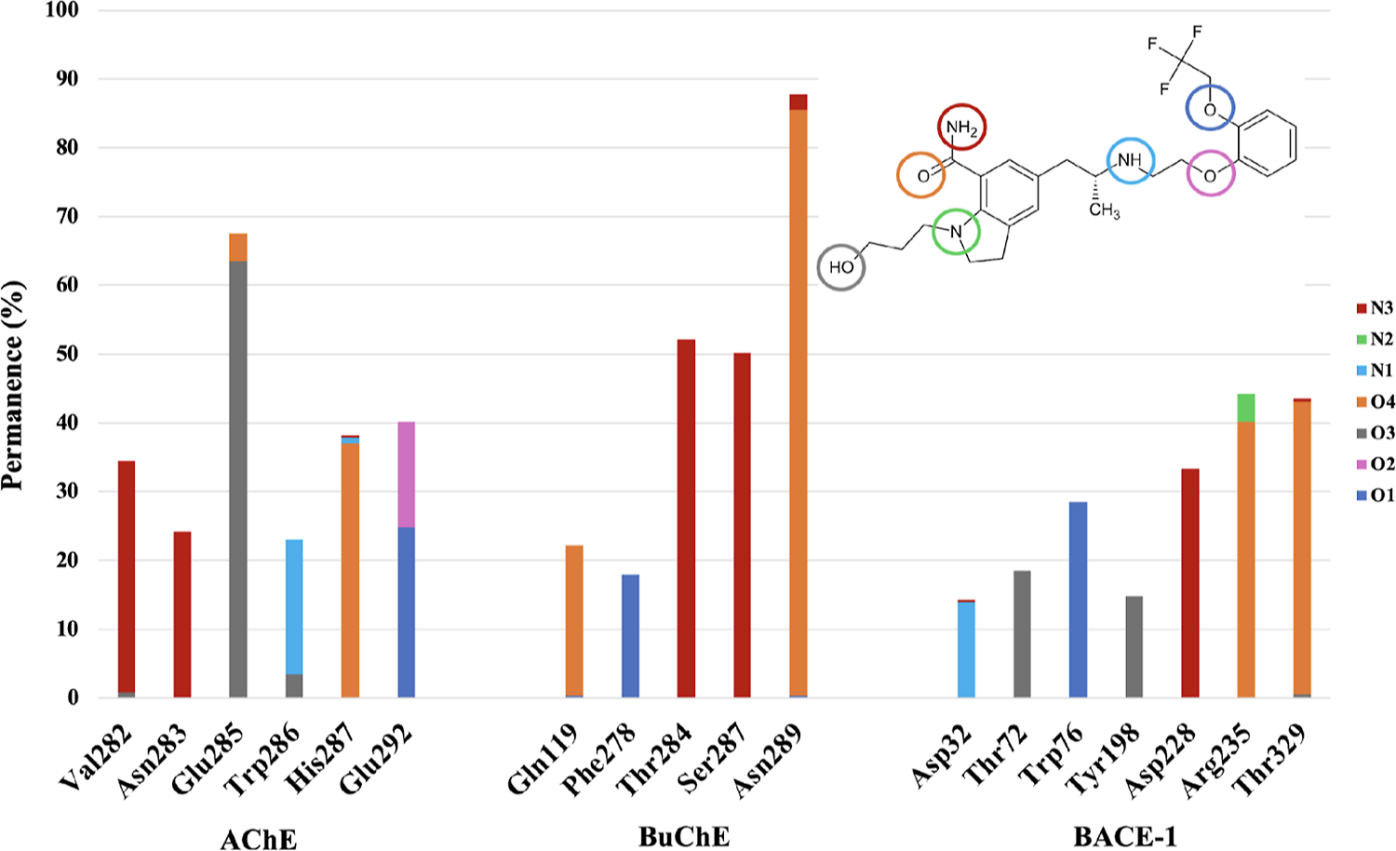
Permanence rate of hydrogen interactions (H-bond) of silodosin in the active site of AChE, BuChE, and BACE-1 during the productive phase of MD simulations and identification of the involved pairs.

Silodosin establishes hydrogen bonding during most of the productive phase between the hydroxyl oxygen from the side chain and Glu285 (63.5%) at the AChE active-binding site. In addition, Trp286 interacts with the tertiary amine from the ligand (19%); nitrogen from amide linked to the indoline group establishes hydrogen bonds with Val282 (33.6%) and Asn283 (24.1%) and the oxygen attached to the benzyl ring with Glu282 (24.8%). In turn, at the BuChE active site, there are important hydrogen bonds established between the oxygen linked to the indoline group and Gln119 (21.9%) and Asn289 (85.2%) and the nitrogen from the same group and Thr284 (52.1%) and Ser287 (50.1%). A hydrogen bond was also observed between oxygen from silodosin hydroxyl and Phe278 (17.9%). In both cholinesterases, it is possible to notice that the residues involved in long permanence hydrogen bonds are residues located in the peripheral region of active sites,^67^ which suggests that the ligand can compete with the entrance of the substrate into the active site. However, several studies have shown that those interactions are not enough to stabilize complexes with AChE, with interactions between ligand and residues located at gorge and at catalytic areas being required.^68,69^

At the BACE-1 active site, hydrogen bonds were observed during more than 10% of the productive phase with catalytic dyad, between nitrogen from tertiary amide and Asp32 (14.0%) and nitrogen from formamide and Asp228 (33.3%). Those interactions are reported on previous studies with BACE-1 inhibitors, which suggests that silodosin can inhibit its catalytic activity,^70,71^ as shown in the cellular assays (Section 3.3). Residues located in the flap region are also involved in hydrogen bonding, as Thr72 and hydroxyl oxygen (18.5%) and oxygen attached to benzyl ring and Trp76 (28.5%). In addition, hydroxyl oxygen is involved in the hydrogen bond with Tyr198 (14.8%), and the oxygen from formamide interacts with Arg235 (40.2%) and Thr329 (42.5%).

Although hydrogen bonds are important for system stabilization, they are not the only interactions responsible for the maintenance of the structures and for the biological response. It is important to evaluate interactions of other nature that can be established between a ligand and protein. For that purpose, representative structures were chosen for each system, showing the conformation at the time 190.45 ns for ACHE–Silodosin, 78.25 ns for BuChE–Silodosin, and 88.3 ns for BACE-1–Silodosin (Figures 10, 11, and 12).

**Figure 10:**
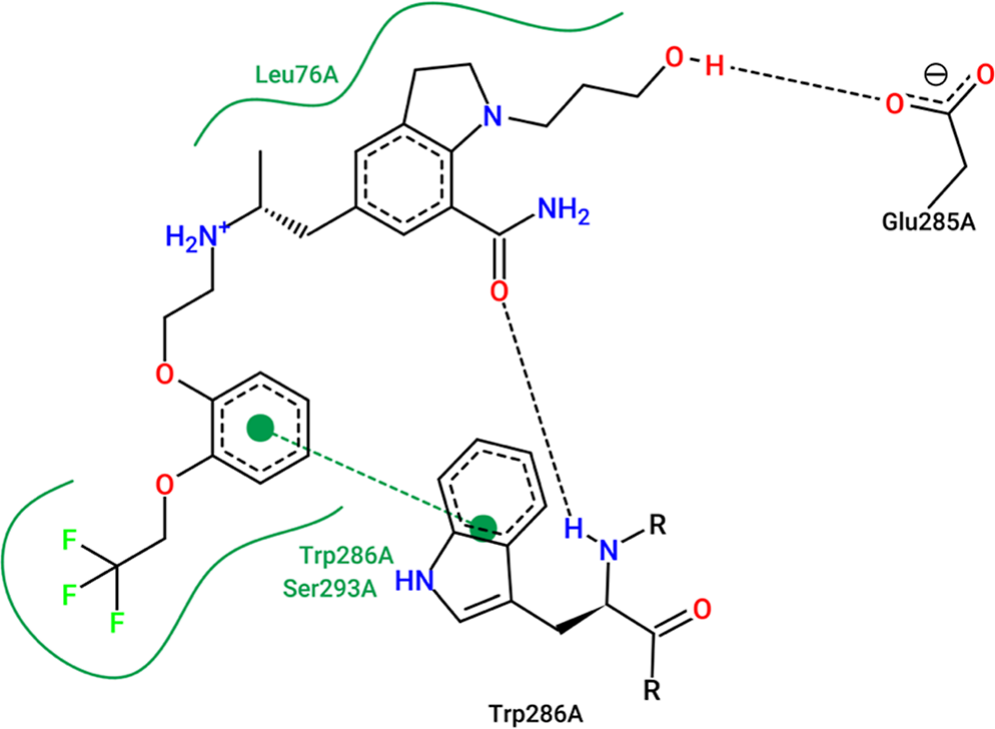
Interactions of silodosin at the AChE binding site obtained from the MD simulation representative structure (*t* = 190.45 ns). Green curved lines represent hydrophobic interactions; green dashed lines represent pi-stacking interaction; black dashed lines represent hydrogen bonds.

**Figure 11:**
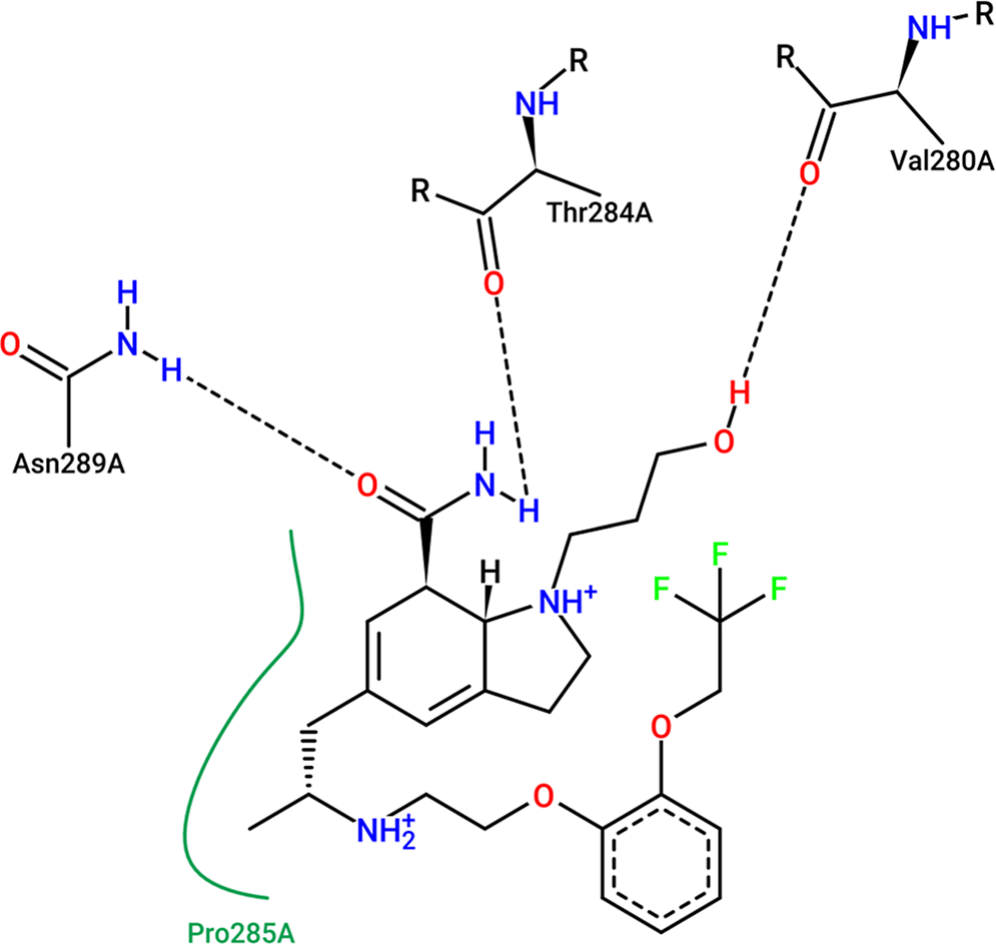
Interactions of silodosin at the BuChE binding site obtained from the MD simulation representative structure (*t* = 85.25 ns). Color schemes are identical to Figure 10.

**Figure 12:**
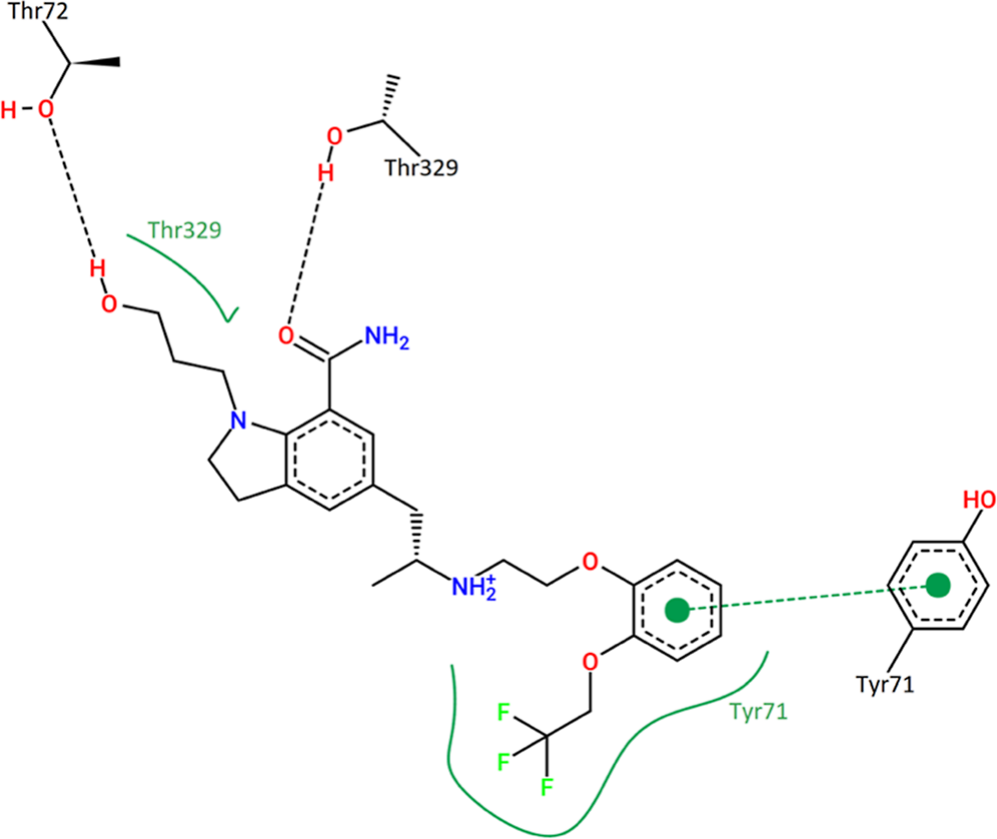
Interactions of silodosin at the BACE-1 binding site obtained from the MD simulation representative structure (*t* = 88.3 ns). Color schemes are identical to Figure 10.

The 2D representative interaction map of silodosin complexed to AChE shows that the trifluoroethyl group is capable of establishing hydrophobic interactions with Trp286 and Ser293. The catecholic group also interacts with Trp286 through pi-stacking, and this interaction seems to be important for AChE inhibition.^72,73^ The indoline group also establishes hydrophobic interactions with Leu76. There can also be observed two hydrogen bonds between the hydroxyl from the indoline side chain and Glu285 and between the oxygen from the formamide group and the Trp286 residue. Although silodosin is capable of establishing interactions that were reported in previous studies, it is important to emphasize that the residues demonstrated at the representative structure correspond to residues that are located at the peripheral site. Previous studies have shown that those interactions are important for inhibition of cholinesterases^74^ but if there is no interaction in the gorge, the ligand–macromolecule complex should not stabilize. This fact could be related to the low affinity for AChE.

The representative structure interaction map of silodosin complexed to BuChE shows that the indoline group establishes hydrophobic interactions with Pro285, and those interactions have been described in previous in silico studies with BuChE inhibitors.^75,76^ Besides hydrophobic interactions, it can be observed that oxygen from the formamide group interacts with Asn289, an interaction that was demonstrated to occur during more than 80% of simulation (Figure 7). This interaction also has been reported as important to BuChE inhibition, since it is related to maintaining the inhibitor inside the site and blocking the substrate interaction.^77^ Additionally, the amine from the formamide group establishes hydrogen bonds with Thr284, and the hydroxyl group from the indoline side chain interacts with Val280 also through hydrogen bonds.

The representative interaction map of silodosin complexed with BACE-1 illustrates the hydrogen bonds with residues Thr72 and Thr329, while Thr329 also establishes hydrophobic interactions with the side chain. Additionally, the silodosin catechol ring establishes pi-stacking interactions with Tyr71, and the trifluoroethanol group attached to the ring also establishes hydrophobic interactions with Tyr71. Residues Tyr71 and Thr72 are considered relevant for BACE-1 inhibitor development, since they are located at the flap loop and are responsible for conformational maintenance and catalysis structure,^65^ which reveals that the silodosin triggers interactions capable to inhibit BACE-1 catalytic activity.

It is important to notice that, in comparison to previous docking studies involving silodosin and AChE, BuChE, and BACE-1,^29^ the interactions changed significantly due to the consideration of protein flexibility. BuChE complex simulation revealed the occurrence of long permanence interactions that are fundamental to BuChE inhibition. To BACE-1, despite molecular docking showing interactions with the catalytic Asp32 while the representative structure of MD did not, the analysis of hydrogen bonding showed that both Asp32 and Asp128 are involved in long permanence interactions that suggests potential to inhibit the activity of the enzyme (Figure 9).

In addition to the significance of intermolecular interactions, binding free energies play a crucial role in driving molecular processes such as molecular association and chemical reactions.^78^ The strength of biomolecular interactions, such as the ones involved in the recognition of an inhibitor, can be quantified by the evaluation of their free energy.^79^ For this reason, it was important to evaluate the thermodynamic aspects of complexes between silodosin and AChE, BuChE, and BACE-1. Since the docking programs simplify energy functions and fail to consider the flexibility of proteins,^80^ the Poisson–Boltzmann molecular mechanics (MM/PBSA) method was applied to productive phases of simulations of complexed systems (Table 1).

**Table 1:** Binding Free Energy and Components Calculated by g_mmpbsa Tools

system	*E*_vdW_ (kJ/mol)	*E*_elec_ (kJ/mol)	*G*_MM_ (kJ/mol)	*G*_polar_ (kJ/mol)	*G*_nonpolar_ (kJ/mol)	Δ*G*_binding_ (kJ/mol)
AChE–silodosin	–0.001	0.046	0.045	–1.313	–3.854	–1.489
BuChE–silodosin	–167.952	–24.197	–192.149	115.276	–4.224	–93.422
BACE-1–silodosin	–24.429	–4.179	–28.608	15.106	1.605	–15.768

The MM/PBSA method is a versatile approach that allows the estimation of binding free energies and achieves a good balance between reliability of results and efficiency.^81^ By using this method, it was observed that the binding free energy of AChE complexed to silodosin corresponds to −1.489 kJ/mol and the van der Waals interactions are not significant for the complex stabilization. Those results illustrate that the association of a ligand and protein is not prone to occur and corroborate to what was observed at in vitro tests, in which silodosin did not show great ability to inhibit AChE catalytic activity. Furthermore, previous studies on reference molecules with inhibitory activity against AChE, such as galantamine and rivastigmine, have reported higher binding free-energy values and greater contributions from van der Waals interactions.^82^ This finding is supported by the residue decomposition analysis (Figure 13), which indicates that individual residue contributions were insufficient to stabilize the complex, and the residues with the highest energy contributions were located outside the binding site.

**Figure 13:**
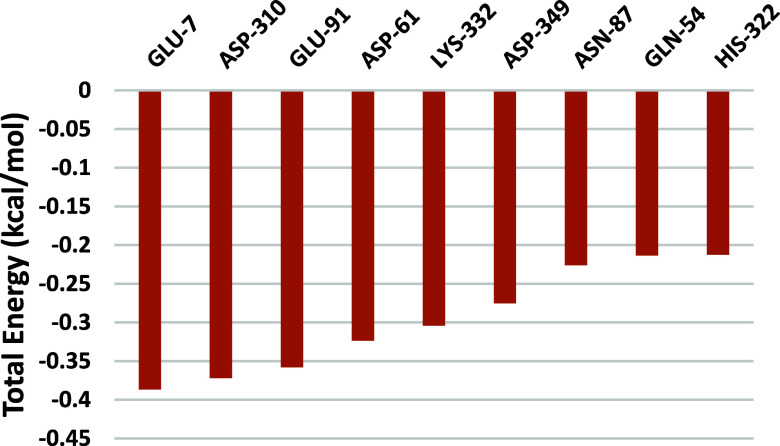
Per-residue decomposition of the total binding free energy for the silodosin–AChE complex, calculated using the MM/PPBSA method during the productive phase (Δ*G* <−0.2 kcal/mol).

In contrast, the binding free energy of the BuChE–silodosin complex is −93.422 kJ/mol (Table 1). This value shows that this compound can create a more stable system with BuChE than donepezil and other active compounds. The van der Waals interactions play an essential role in this process, as also observed in previous studies,^83,84^ and corroborate the inhibition values observed in vitro. In addition, residue decomposition analysis (Figure 14) supports interactions observed in Figure 11 which are likely to occur and contribute to complex stabilization, since residues such as Pro285 and Val280 showed significant energy contribution. Other major contributions are also associated with active site residues, as Trp82, Leu286, Val288, and Tyr332.

**Figure 14:**
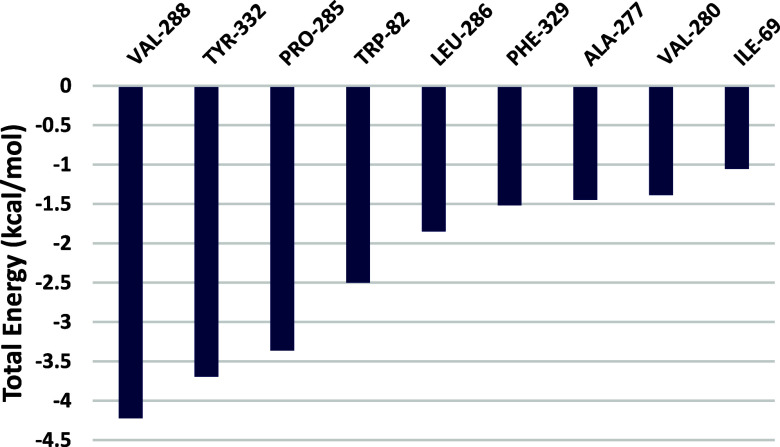
Per-residue decomposition of the total binding free energy for the silodosin–BuChE complex, calculated using the MM/PPBSA method during the productive phase (Δ*G* <−1.0 kcal/mol).

For the BACE-1–silodosin complex, the binding free energy is −15.768 kJ/mol (Table 1), illustrating the van der Waals interactions and hydrogen-bonding importance for stabilization, and corroborates the long permanence interactions observed in Figure 9. Those results are in line with previous molecular dynamics studies of BACE-1 inhibitors^85,86^ and confirm that silodosin presents the requirements to inhibit BACE-1 activity. The residue decomposition analysis (Figure 15) indicates that individual residue contributions were insufficient to stabilize the complex, but even with this limitation, catalytic residue Asp32 and other important residues for BACE-1 catalysis as Tyr71 showed the highest energy contribution.

**Figure 15:**
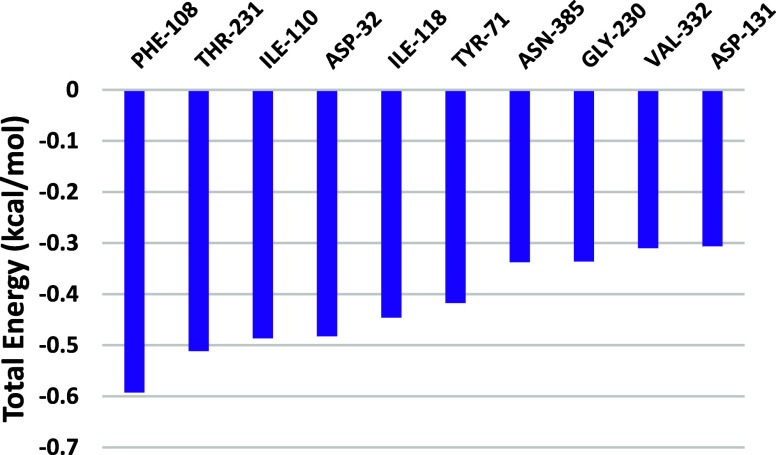
Per-residue decomposition of the total binding free energy for the silodosin–BACE-1 complex, calculated using the MM/PPBSA method during the productive phase (Δ*G* <−0.3 kcal/mol).

Despite previous studies presenting silodosin as a potential triple inhibitor,^43^ in vitro tests and MD simulations demonstrated its selectivity to BuChE over AChE and its ability to inhibit BACE-1 activity in low concentrations. Those results suggest that silodosin can be useful in the treatment of advanced stage AD, since BuChE activity is increased in advanced patients as a mechanism to compensate decreasing AChE levels related to the deposition of amyloid plaques.^87,88^ We intend to use the results obtained to guide the design of new derivatives with more efficient triple inhibition of AChE, BuChE, and BACE-1.

## 4 Conclusions

In vitro and in silico studies were used to evaluate silodosin, a potential triple inhibitor of AChE, BuChE, and BACE-1 previously identified through hierarchical virtual screening, as a candidate for the treatment of AD. Enzymatic assays were performed, and despite the low values of AChE inhibition, silodosin showed a great inhibitory profile against BuChE and BACE-1, in concentrations low enough to be considered safe, according to cellular viability assays (3 μM). The results of MD simulations corroborate what was observed in vitro*,* where silodosin complexed with BuChE and BACE-1 showed stabilization in the early stages of simulation, while the system silodosin–AChE showed late stabilization. Finally, the treatment of DA is still insufficient to stop the disease’s progress. Thus, this study was performed under rational drug design strategies, which were applied to drug repurposing approach and in vitro and in silico studies, and the results suggest silodosin can be used as a potential molecular scaffold for multitarget inhibition in AD. However, further optimization in its structure and tests are needed.
